# Evaluation and analysis of brittleness and acoustic emission characteristics of tight sandstone under the influence of acid-treatment

**DOI:** 10.1038/s41598-026-45184-y

**Published:** 2026-04-07

**Authors:** Weile Geng, Shengli Guo, Gun Huang, Jun Wang

**Affiliations:** 1 School of Safety and Management Engineering, Hunan Institute of Technology, Hengyang, 421002 China; 2 State Key Laboratory of Coal Mine Disaster Dynamics and Control, School of Resources and Safety Engineering, Chongqing University, Chongqing, 400030 China; 3 SINOPEC Geophysical Research Institute Co., Ltd, SINOPEC, Nanjing, 211103 China

**Keywords:** Brittleness, Acoustic emission, Radial strain response, Acid treatment, Tight sandstone reservoirs, Energy science and technology, Engineering, Materials science, Solid Earth sciences

## Abstract

Acid treatment has been proven to effectively reduce reservoir fracture pressure, but the variation pattern of sandstone brittleness after acid treatment remains unclear. To effectively evaluate the characteristics of brittleness changes, uniaxial compression tests were conducted on tight sandstone subjected to acid treatment for different durations, and a brittleness index evaluation method based on radial strain response was established. The results show that sandstone uniaxial compressive strength decreases stepwise with prolonged acid treatment. Elastic modulus decreases slowly initially, drops sharply after 24 h of acid treatment, and stabilizes between 120 and 168 h. Poisson’s ratio decreases linearly after 6 h of acid treatment. The brittleness index first increases and then decreases, peaking at 12–24 h of acid treatment—consistent with AE results showing more high-intensity signals during the elastic compression stage of axial stress-strain curves. After acid treatment, sandstone crack initiation stress first increases slowly, then gradually decreases, and finally drops rapidly. This is attributed to acid-rock reaction-induced dissolution of minerals and cementing materials, which detaches fine-grained matrix particles, increases porosity, and reduces crack initiation stress. The findings provide a reference for engineering design and construction of high fracture pressure tight sandstone reservoirs.

## Introduction

Tight sandstone gas reservoirs typically require large-scale fracturing to achieve stable single-well production and maintain effective production periods^1,2^. However, due to the deep burial and ultra-tight nature of tight sandstone reservoirs, the fracture pressure of the reservoir is often high, making it difficult to fracture the reservoir^3^. For deep-buried tight sandstone reservoirs, acid pre-treatment is generally used to weaken the mechanical properties of the surrounding rock near the wellbore^4,5^, thereby reducing the reservoir’s fracture pressure. The dissolution of soluble minerals and cementing materials during the acid pre-treatment process may alter the brittle failure characteristics of the rock^6–8^. The brittleness characteristics of the rock are crucial indicators for reservoir fracturing^2,9,10^, as they can be used to quantitatively assess the complexity of the fracture network after reservoir modification^11^. Therefore, a reasonable and accurate evaluation of the rock’s brittleness after acid treatment is of great significance for the fracturing of tight sandstone reservoirs.

Acid treatment leads to a reduction in the mechanical strength of the rock^12^. Due to differences in mineral content and cementation strength, different rock types exhibit varying changes in mechanical properties after acid treatment, and their acid-etched surfaces display varying degrees of dissolution and etching^13^. The dissolution of soluble minerals by acid increases rock porosity^7^, and the surface porosity of the rock shows significant fractal characteristics^14^. For rock samples with ultra-low porosity, acid solution reamain difficult to penetrate into the core under pump pressure^15^, indicating that the acid-rock reactions mainly occur on the rock surface. After treatment with hydrochloric or sulfuric acid solutions, the brittleness of sandstone weakens and gradually exhibits plastic failure characteristics. As the pH of the acid solution and the duration of acid treatment increase, the peak stress, elastic modulus, and Poisson’s ratio of sandstone decrease gradually^6^. Existing research shows that acid treatment has a significant deteriorating effect on the physical and mechanical properties of rocks, which strongly influences rock brittleness. Moreover, acid-rock reactions exhibit obvious time dependency^16^. The mechanical degradation of rock under chemical exposure is not instantaneous, but evolves with reaction time, involving complex time-dependent chemo-mechanical coupled processes^17^. This temporal evolution directly influences the constitutive behavior and damage accumulation of the rock^18^. Therefore, to accurately predict and optimize the acidizing operation window in field applications, it is imperative to systematically investigate the time-dependent evolution of rock brittleness under acid treatment, bridging the gap between short-term mechanical property degradation and long-term chemical damage effects.

Generally speaking, the higher the brittleness of reservoir rock, the more favorable it is for the formation of fractures, which tend to form a continuous and interconnected fracture network. Conversely, when the reservoir rock exhibits more pronounced plastic characteristics, the resulting fracture morphology becomes simpler^19,20^. Due to the differences in the concept of brittleness and the factors considered across various disciplines, there is currently no unified standard for defining brittleness, nor is there an exact formula for calculating a brittleness index^21–24^. Rickman, et al.^25^ suggested that elastic modulus and Poisson’s ratio are the main parameters influencing rock brittleness and proposed a rock brittleness index based on these two parameters. However, the rock brittleness index obtained by this method lacks sufficient physical significance, and the brittleness evaluation results are highly dependent on the selection of elastic modulus and Poisson’s ratio. Jarvie, et al.^26^ argued that rock brittleness is related to the content of brittle minerals within the rock and proposed different brittleness evaluation models that account for the contribution of various brittle minerals to rock brittleness^27,28^. However, this method overlooks the impacts of the rock’s depositional history and the in-situ stress environment.

Brittleness, as an important mechanical indicator of rock, reflects the strength and deformation characteristics during the rock failure process. Scholars have proposed corresponding brittleness index calculation formulas based on the rock’s strength parameters and deformation parameters^29,30^, as well as brittleness evaluation models based on the complete stress-strain curve^31^. However, this approach overlooks the instant drop in the stress-strain curve at the moment of brittle rock failure, making it difficult to capture the post-peak stress-strain curve of the rock. The process of rock deformation and failure under load essentially reflects the accumulation and dissipation of energy. Zhang et al.^32^ and Li et al.^33^ proposed a brittleness index evaluation model based on energy evolution, which considers the influence of energy both before and after the peak on rock brittleness. However, this method also overlooks the instant drop in stress after the peak in brittle rocks, making it difficult to obtain the fracture energy of the rock in the post-peak stage.

Currently, significant progress has been made in the study of rock brittleness. However, research on the effects of acid treatment of varying durations on the brittleness of sandstone is relatively limited. Brittle failure in rocks often occurs at low strain states. Microcracks within the rock initiate, propagate, and eventually form multidimensional fracture planes through unstable coalescence under external forces. The initiation of microcracks within the rock can be determined by the crack initiation stress, which can be derived from the rock’s stress-strain curve. However, in brittle rocks, the stress-strain curve drops sharply after reaching the peak, making it difficult to obtain the complete stress-strain curve for the sample. In fact, the pre-peak portion of the stress-strain curve primarily reflects the elastic and inelastic deformation properties of the rock, while the post-peak portion mainly reflects the rock’s load-bearing capacity after failure. Reservoir modification in oil and gas fields focuses more on the initiation and propagation of rock cracks, with less emphasis on the post-fracture morphology of the rock. Therefore, establishing a brittleness index evaluation model that considers the stress-strain relationship from the crack initiation stress to the peak stress is more meaningful for unconventional reservoir modification.

## Sample preparation and testing scheme

The samples were taken from the outcrop of the Xujiahe Formation tight gas reservoir. The sandstone is primarily composed of quartz and feldspar, with some clay minerals such as illite and chlorite. The average density of the sandstone is 2.55 g/cm³, with porosity mainly ranging from 2% to 4% and permeability primarily between 0.01 mD and 0.1 mD. According to the standards recommended by the International Society for Rock Mechanics (ISRM), the samples were processed with a height-to-diameter ratio of 2:1, a diameter of 25 mm, and a height of 50 mm. To ensure testing accuracy, the non-parallelism of the sample’s end surfaces was kept within 0.05 mm.

Hydrochloric acid (HCl) and hydrofluoric acid (HF) mixed to form mud acid can effectively dissolve carbonate minerals, clay, and silicates in sandstone reservoirs, weakening the mechanical properties of the reservoir rock and improving its physical characteristics. The acid solution used was a mixture of 12% HCl and 2% HF. The duration of the acid-rock interaction significantly impacts the physical properties of the reservoir rock. During acid treatment, the acid first reacts with carbonates and clay minerals in the rock, increasing its porosity. However, as the acid treatment time increases, the hydrofluoric acid in the mud acid may react with the rock again, forming reaction precipitates on the rock surface, which can reduce its porosity and permeability. Additionally, prolonged acid pre-treatment can lead to the collapse of the near-wellbore area. Therefore, it is essential to reasonably control the duration of the acid pre-treatment. The prepared samples were subjected to acid pre-treatment for different durations: 1 h, 2 h, 4 h, 6 h, 12 h, 24 h, 48 h, 72 h, 120 h, and 168 h. The sample codes were H1, H2, H4, H6, H12, D1, D2, D3, D5, and D7, respectively. The selection of acid treatment durations was based on a combination of preliminary exploratory tests and the typical time scales of acid-rock reactions in laboratory studies and field operations. The control group, which was not subjected to acid treatment, was coded as H0. For each treatment condition, two parallel specimens prepared and tested separately to ensure the reproducibility and statistical significance of the results. After the uniaxial compression test, a high-precision balance was used to measure the mass changes of the samples before and after acid treatment.

**Fig. 1:**
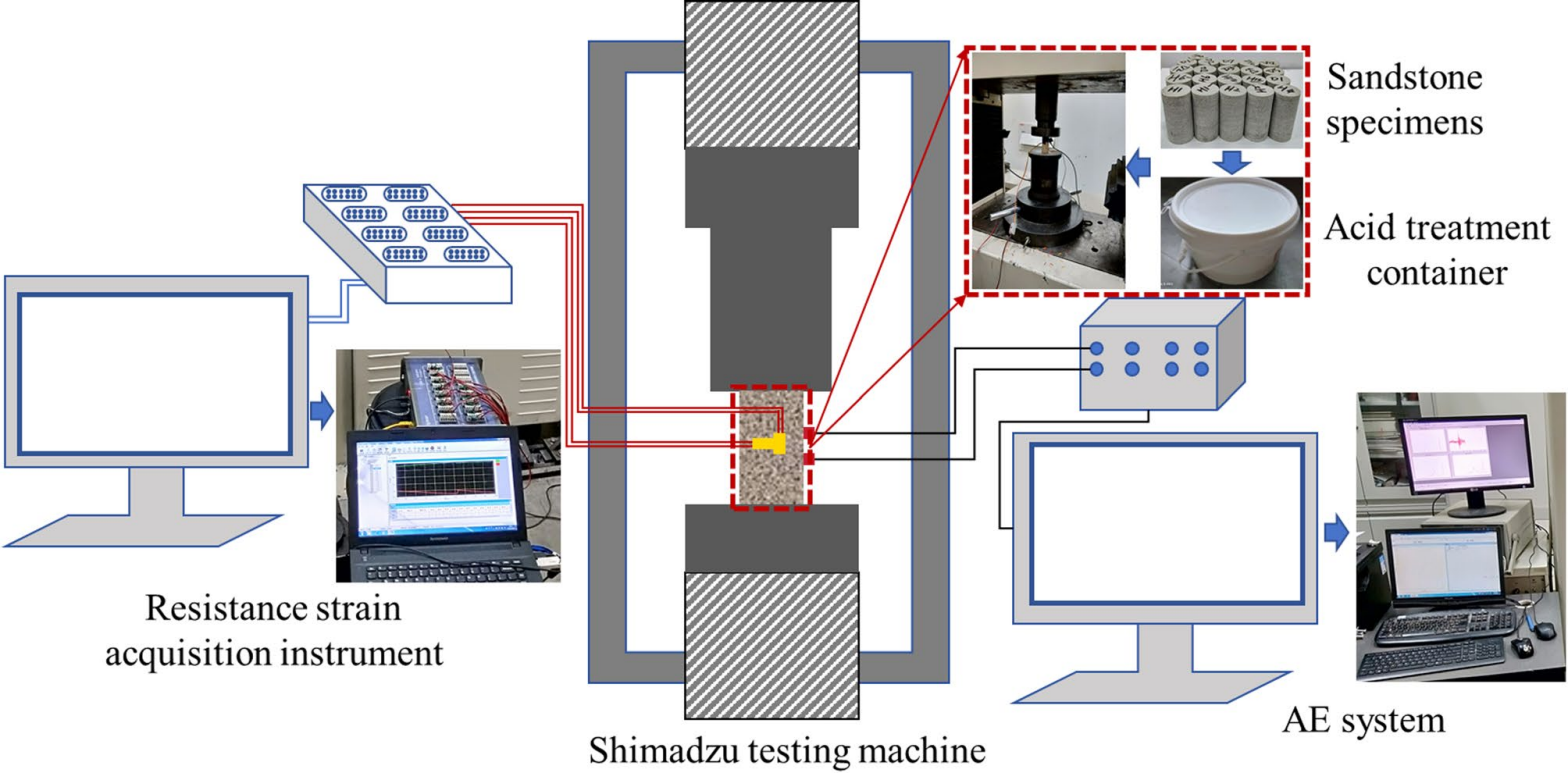
Test system and equipment.

All tests were conducted using a Shimadzu testing machine with displacement-controlled loading at a loading rate of 0.1 mm/min. During the tests, a PCI-2 acoustic emission monitoring system was used to monitor the acoustic emission signals of the samples under different acid treatment durations. The acoustic emission parameters were set as follows: preamplifier gain at 40 dB, sampling rate at 1 MSPS, and acquisition threshold at 45 dB. A strain gauge was used to monitor the axial and radial strain changes of the samples during the tests. After the tests, the debris particles that fell from the fracture surfaces under uniaxial compression were collected and weighed. Meanwhile, XRD tests were employed to analyze the changes in mineral composition of sandstone specimens before and after acid treatment. The test system and equipment are shown in Fig. 1.

## Test results and analysis

### Analysis of changes in mineral composition after acid treatment

**Fig. 2:**
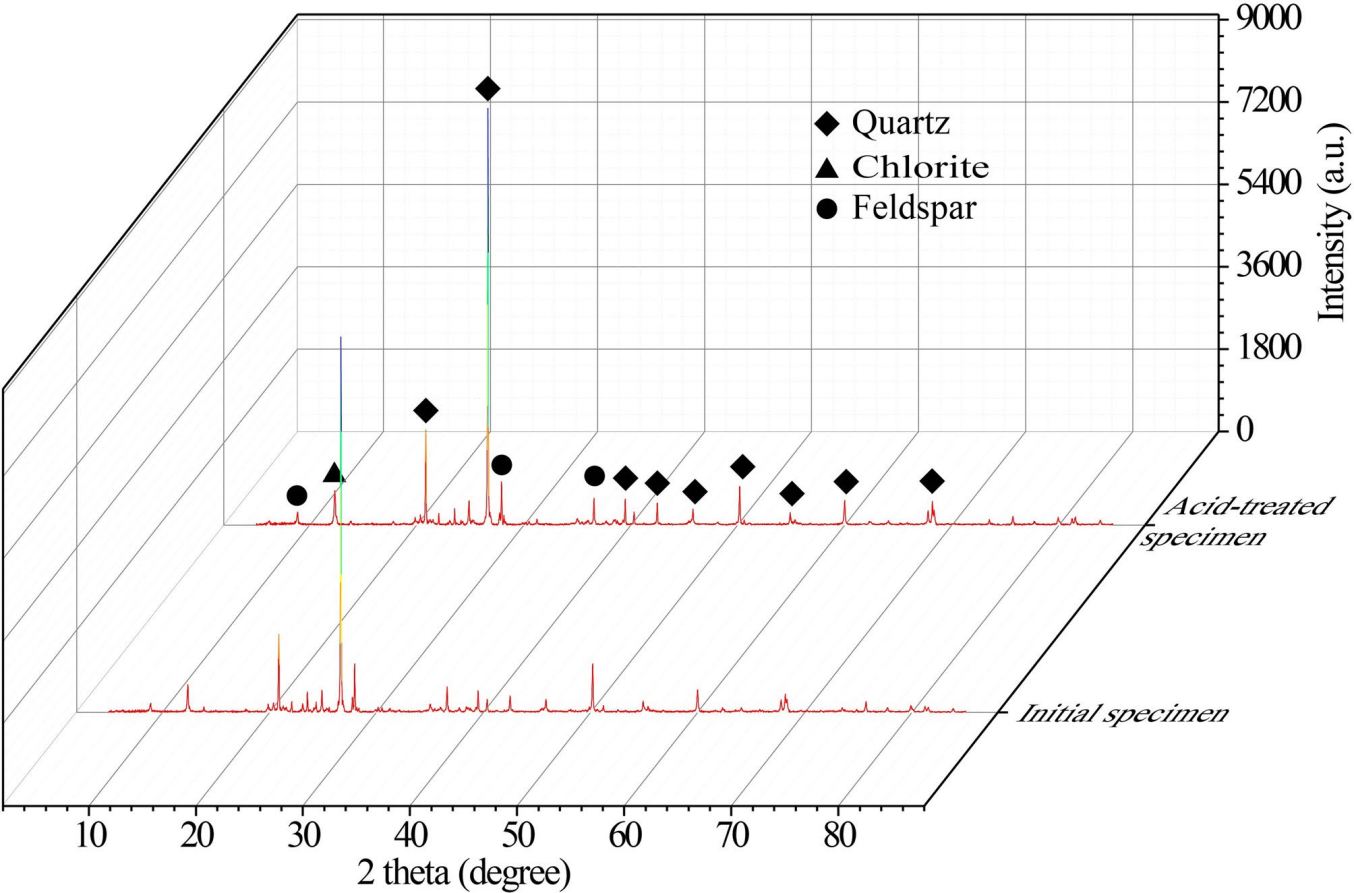
XRD diffraction patterns of initial specimen and acid-treated specimen.

The XRD patterns of sandstone before and after acid treatment are shown in Fig. 2. According to the X-ray diffraction results, the mineral composition of tight sandstone is dominated by quartz, feldspar, and chlorite. After acid treatment, the diffraction peak intensities of quartz, feldspar, and chlorite in the sandstone minerals all decreased to varying degrees, indicating that the acid treatment dissolved some sandstone minerals. Existing studies have shown that reservoirs in this area are dominated by calcareous and siliceous cementation^34^. After acid treatment, reservoir pores are mainly composed of primary intergranular pores between debris particles and intragranular dissolved pores generated by dissolution.

### Uniaxial compression curve

**Fig. 3:**
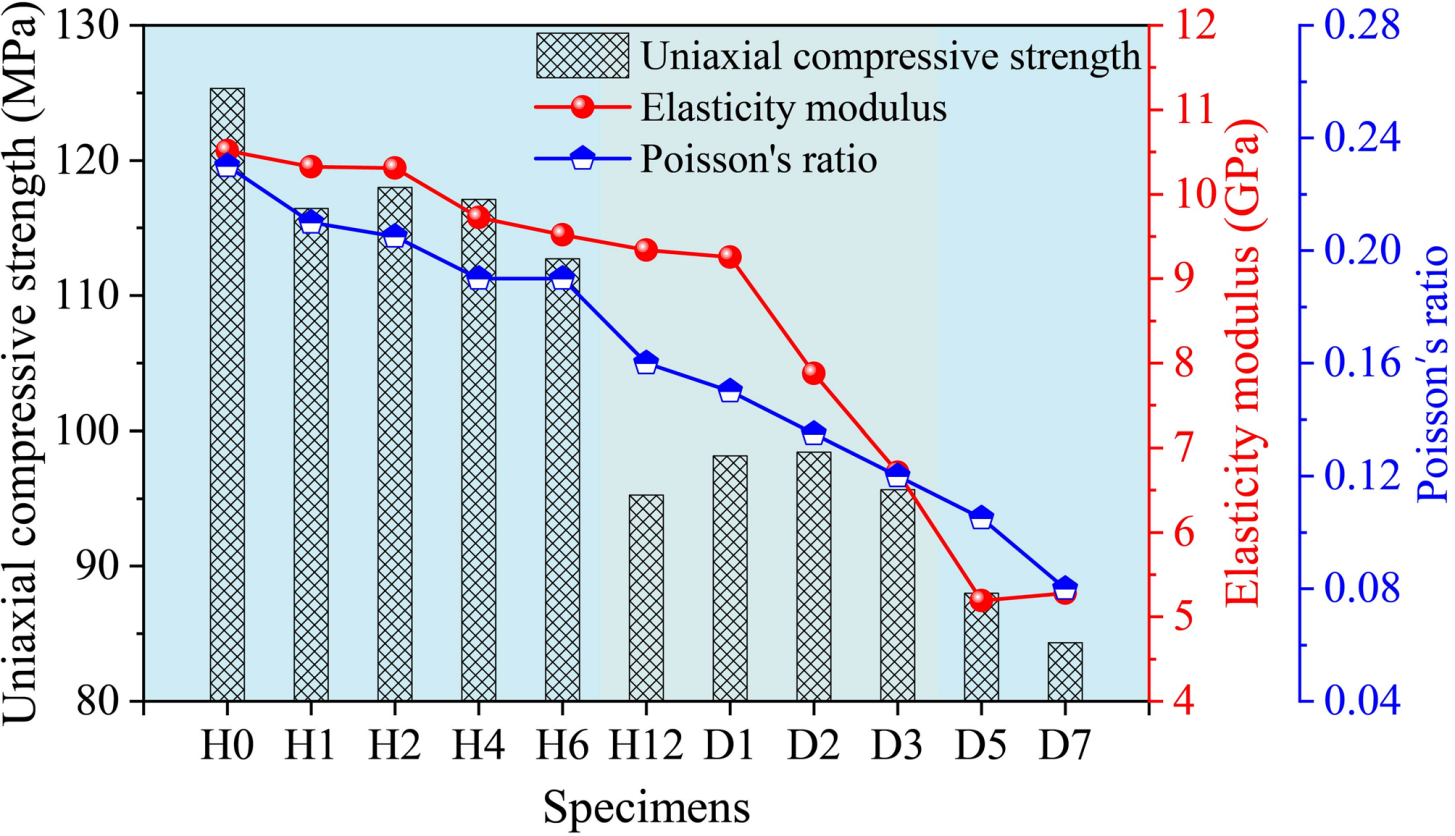
Variations in mechanical parameters of sandstone under uniaxial compression.

The uniaxial compressive strength of sandstone exhibits a stepwise decrease with increasing acid treatment time (Fig. 3). The elastic modulus decreases slowly at first, then drops rapidly after 24 h of acid treatment, and remains essentially stable under the 120-hour and 168-hour acid treatments. Poisson’s ratio remains nearly unchanged during the first 6 h of acid treatment, but decreases approximately linearly thereafter. After 24 h of acid treatment (sample D1), the uniaxial compressive strength, elastic modulus, and Poisson’s ratio of the sandstone decrease by 21.6%, 12%, and 34.8%, respectively. After 168 h of acid treatment (sample D7), these three parameters decrease by 32.7%, 49.8%, and 65.2%, respectively.

### Mass dissolution

**Fig. 4:**
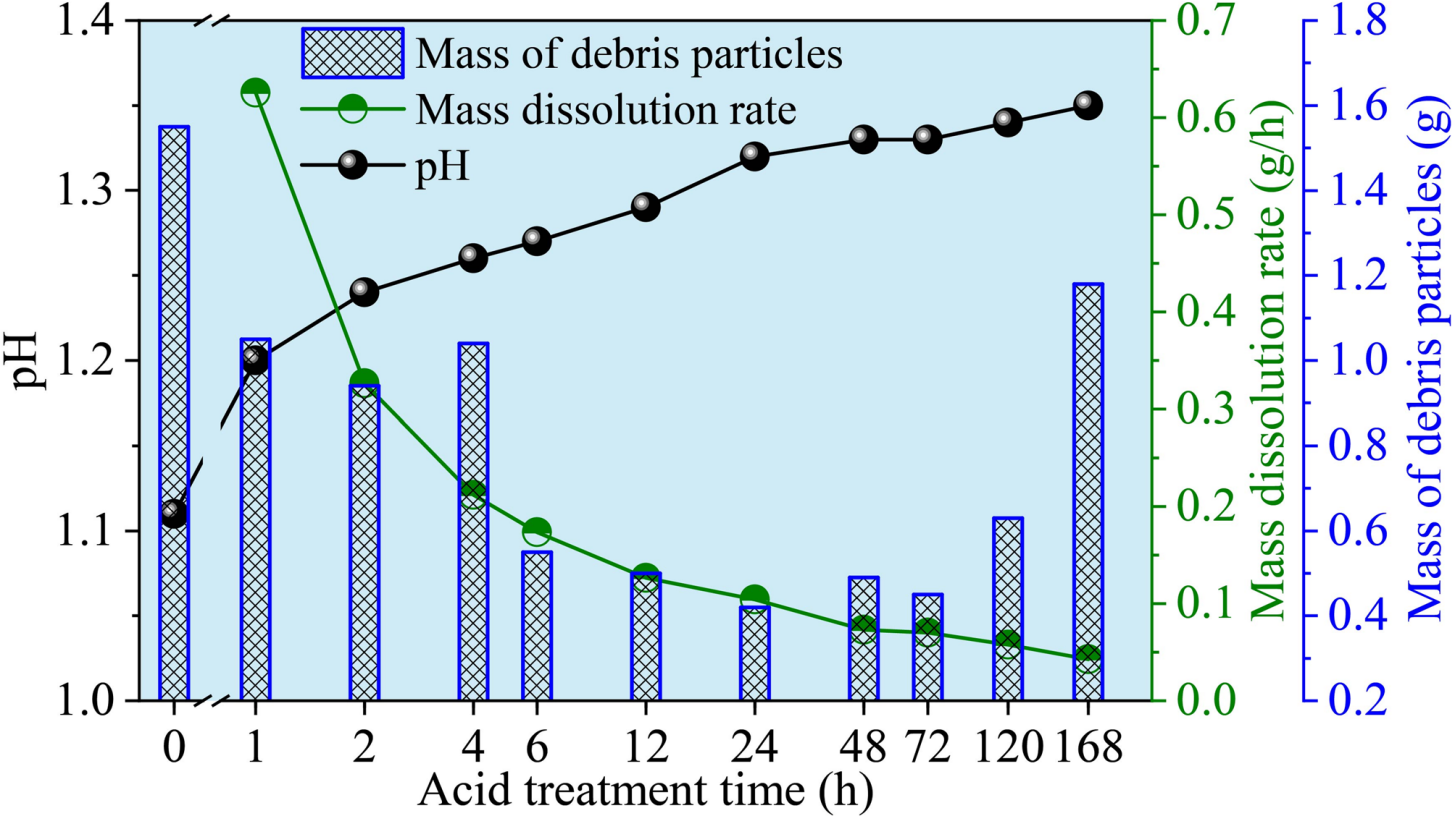
Variations in pH, mass dissolution rate, and debris mass with acid treatment duration.

Figure 4 shows the mass changes of sandstone under different acid treatment durations. Considering the limitation of the contact surface between the acid solution and the sandstone samples, the mass dissolution rate was introduced to characterize the mass loss of sandstone under different acid treatment durations. The mass dissolution rate is defined as the ratio of the mass change of the sample before and after acid treatment to the acid treatment time. Upon initial contact between the sample and the acid solution, the reaction is vigorous, and the mass dissolution rate decreases rapidly. After approximately 24 h of acid treatment, the mass dissolution rate of the samples tends to stabilize. After the uniaxial compression tests, debris particles shed from the bulk fragments were collected and weighed to analyze the effect of acid treatment duration on the mass of detached debris. The results show that the mass of debris particles first decreases and then increases with increasing acid treatment time, and sample D1 exhibits the lowest debris mass after failure. The pH values of the acid solution were also measured at different times during acid treatment, and the corresponding pH evolution over time is presented in Fig. 4. In the early stage of the acid-rock reaction, the pH of the acid solution rises noticeably from 1.11 in the initial solution to 1.32 after 24 h, representing an increase of 18.9%. As the acid-rock reaction proceeds, the change in pH of the acid solution gradually stabilizes.

### Determination of crack initiation stress

Brittleness is an important indicator of the mechanical properties of reservoir rocks and the modification of oil and gas reservoirs. Investigating the evolution of brittleness indices following acid treatment provides critical guidance for determining the optimal acid treatment duration. Common methods for evaluating brittleness in unconventional reservoirs include the mineral composition method, the rock mechanical parameters method, the stress-strain curve method, and brittleness evaluation methods based on energy evolution during rock deformation.

Tight sandstone generally exhibits high uniaxial compressive strength. After reaching the peak strength, the stress drops abruptly, making it difficult to obtain complete stress‑strain curves for the specimens. In practice, the pre‑peak portion of the stress‑strain curve mainly reflects the elastic and inelastic deformation characteristics of the rock, whereas the post‑peak portion primarily represents the load‑bearing capacity of the rock after failure. Reservoir stimulation in oil and gas fields focuses more on the initiation and propagation of rock cracks, with less emphasis on the fracture morphology of the rock after failure.It is generally accepted that the volumetric strain of rock can be expressed as the sum of the elastic volumetric strain and the crack volumetric strain induced by the closure or opening of internal cracks under the same stress state. The volumetric strain and elastic volumetric strain can be calculated using Eq. (1).

1 $$\begin{gathered} \varepsilon _{v} = \varepsilon _{1} + 2\varepsilon _{2} \hfill \\ \varepsilon _{{ve}} = \frac{{1 - 2v}}{E}(\sigma _{1} + 2\sigma _{2} ) \hfill \\ \end{gathered}$$

**Fig. 5:**
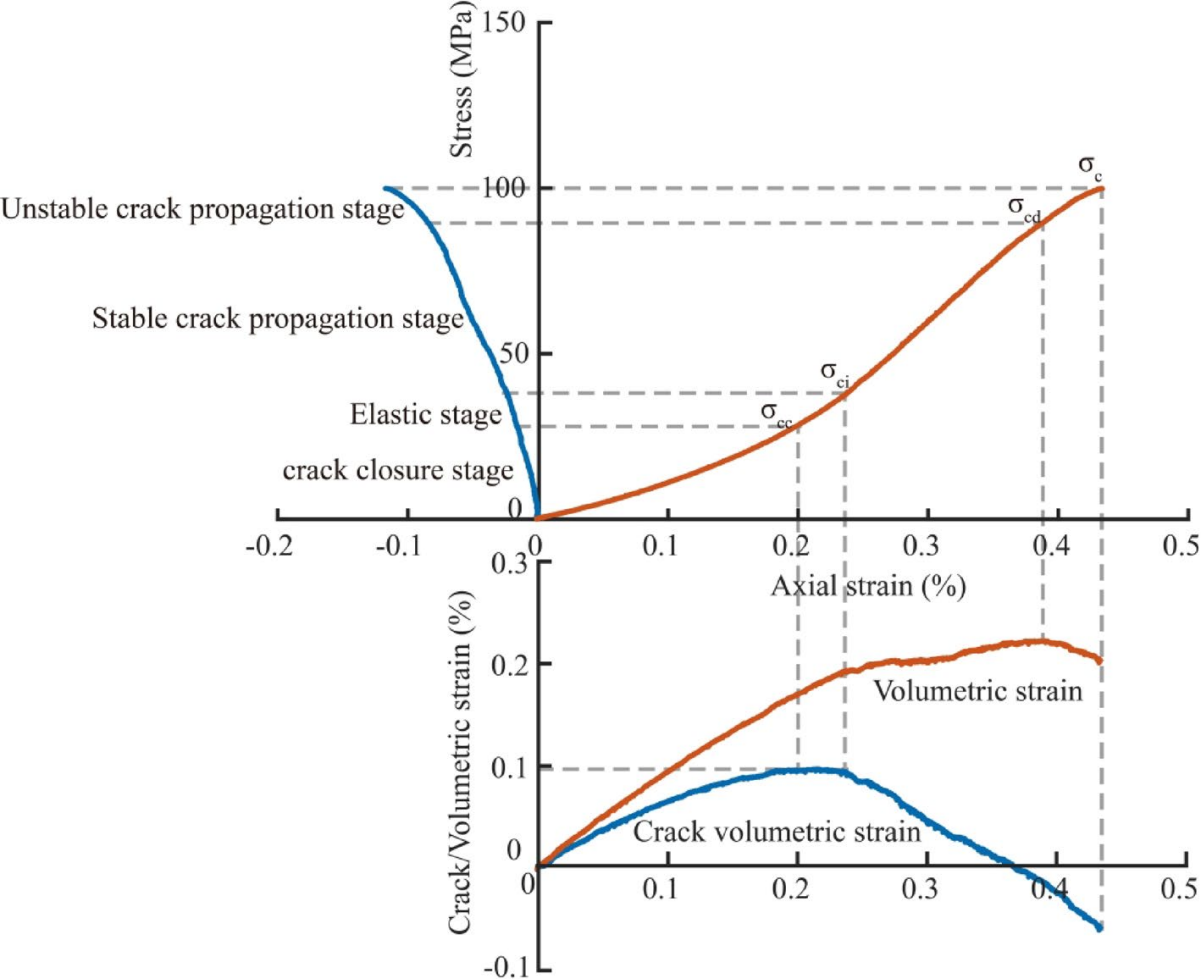
Division of failure stages for brittle rock.

In the equation, $\varepsilon _{v}$ and ${\varepsilon _{{ve}}}$ represent the total volumetric strain and elastic volumetric strain, respectively. By calculating the difference between $\varepsilon _{v}$ and ${\varepsilon _{{ve}}}$ under different stress states, the crack volumetric strain ${\varepsilon _{{vc}}}$, which reflects the closing or opening of internal cracks in the rock during loading, can be obtained. Based on the changes in total volumetric strain and crack volumetric strain in the rock, the pre-peak portion of the stress-strain curve can be divided into four stages, as shown in Fig. 5. When the axial load reaches the crack initiation stress, internal cracks in the rock begin to propagate. With increasing load, crack propagation enters an unstable stage, eventually leading to rock failure.

**Fig. 6:**
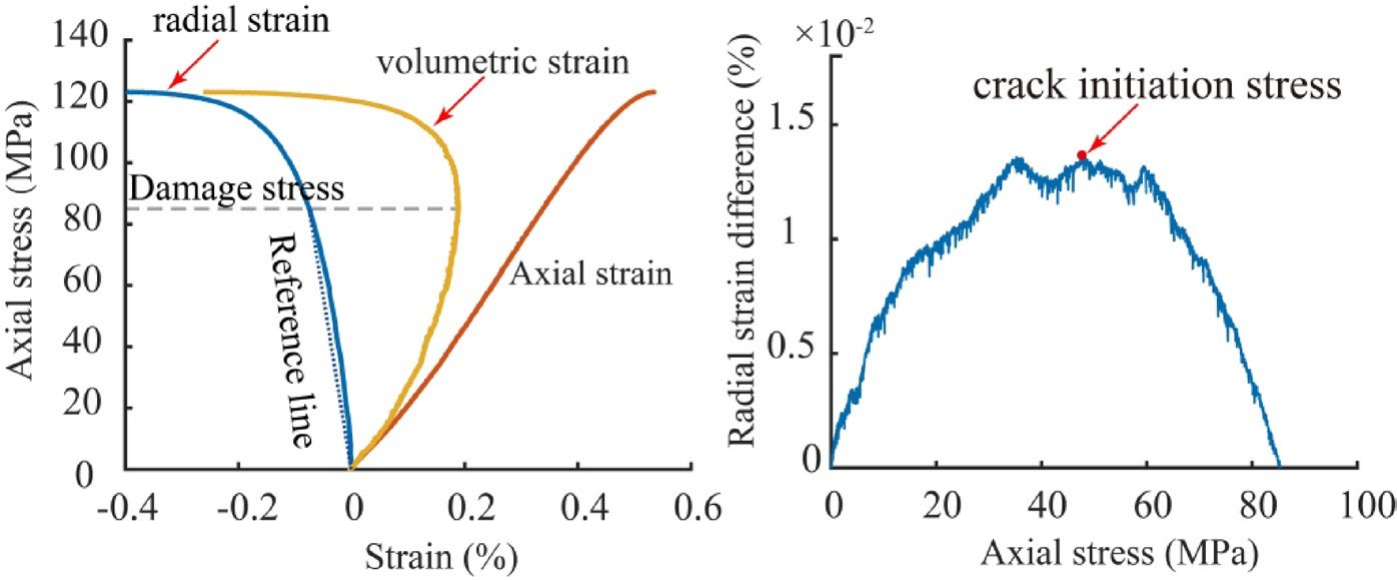
Determination of crack initiation stress.

Therefore, studying rock brittleness indices based on the characteristics of the stress-strain curve before the peak, especially focusing on the stress-strain changes from the onset of crack propagation to peak failure, is of great significance for the modification of unconventional oil and gas reservoirs. Currently, the determination of crack initiation stress mainly relies on the crack volumetric strain method, but this method is highly dependent on the selection of the rock’s elastic parameters. During acid treatment, the dissolution of minerals on the rock surface makes the lateral strain of the sample more sensitive during uniaxial compression, necessitating a more suitable method to determine the crack initiation stress of sandstone samples after different durations of acid treatment. Nicksiar and Martin^35,36^ introduced the radial strain response method in their study of crack initiation stress during the compression of low-porosity rocks. This method first determines the rock damage stress on the axial stress-volumetric strain curve, connects the origin and damage stress points on the axial stress-radial strain curve to form a linear radial strain reference line, and then calculates the difference between the linear radial strain and the measured radial strain. By plotting the relationship between the radial strain difference and axial stress, the peak point is identified, and the corresponding stress at this point is determined as the crack initiation stress (Fig. 6).

### Brittleness index

**Fig. 7:**
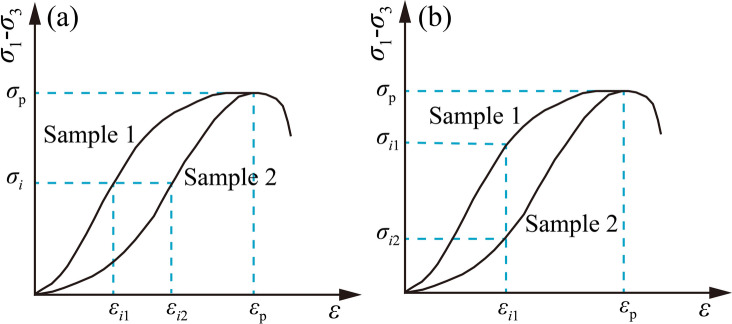
Initial stress and initial strain.

For rocks of different lithologies, multiple corresponding crack initiation strains or crack initiation stresses may exist at the same crack initiation stress or crack initiation strain. As illustrated in Fig. 7, Sample 1 exhibits a distinct plastic yield stage before reaching the peak, while the stress-strain curve of Sample 2 shows an approximately linear variation prior to the peak. According to the energy evolution law of rock under uniaxial compression, Sample 2 accumulates a higher proportion of elastic strain energy in the pre-peak stage compared to Sample 1. After reaching the peak load, Sample 2 fails rapidly, demonstrating stronger brittleness. Therefore, the trend of the pre-peak stress-strain curve exerts a significant impact on determining rock brittleness, and the calculation of the brittleness index needs to comprehensively consider the stress-strain relationship in the range from crack initiation stress to peak stress. Based on the existing brittleness index evaluation method using the complete stress-strain curve, a brittleness index *B*_*i*_ considering the pre-peak crack initiation stress is established as shown in Eq. (2).

2 $${B_i}=\frac{{{\sigma _{c}} - {\sigma _i}}}{{{\sigma _{c}}}} \cdot \frac{{{\varepsilon _{c}}}}{{{\varepsilon _{c}} - {\varepsilon _i}}}$$

In the formula, ${\sigma _{c}}$ represents the peak stress, ${\sigma _i}$ represents the crack initiation stress, ${\varepsilon _{c}}$ represents the peak strain, and ${\varepsilon _i}$ represents the crack initiation strain.

**Fig. 8:**
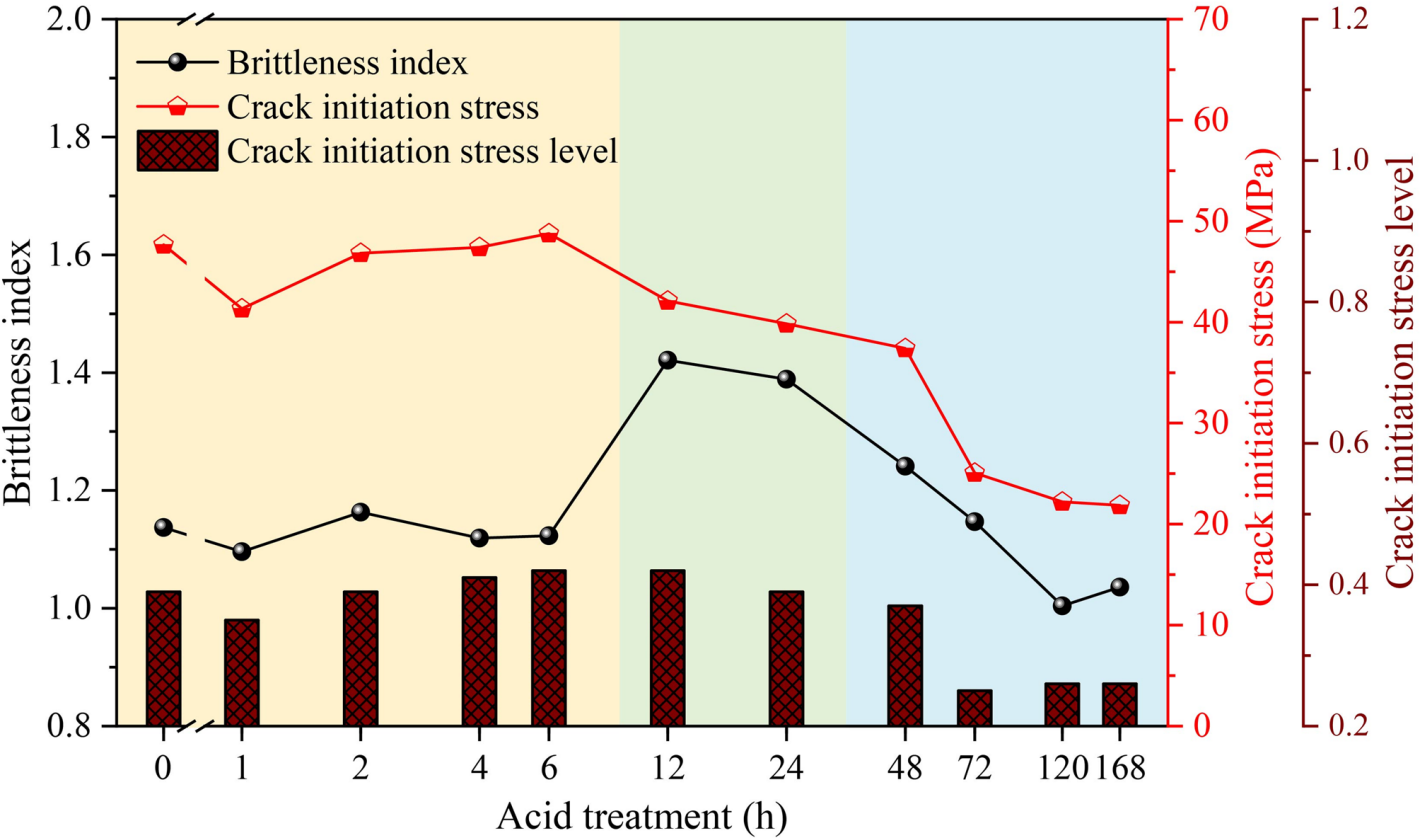
Variation of brittleness index and crack initiation stress with acid treatment times.

Figure 8 illustrates the changes in brittleness index and crack initiation stress with increasing acid treatment time. As the acid treatment time increases, the brittleness index exhibits a trend of initially increasing and then decreasing, reaching its maximum after 12–24 h of acid treatment. The crack initiation stress shows a variation pattern similar to that of the peak strength of the samples. The crack initiation stress level *k* is defined as the ratio of the crack initiation stress to the peak stress, as shown in Eq. (3).

3 $$k=\frac{{{\sigma _{{c}i}}}}{{{\sigma _{c}}}}$$

The crack initiation stress level *k* reflects the heterogeneity and structural differences of the rock sample. A smaller *k* value indicates greater heterogeneity. The mineral composition and grain structure of the rock determine its crack initiation stress. As shown in Fig. 8, with increasing acid treatment time, the crack initiation stress level of the sandstone samples generally exhibits a trend of initially increasing slowly, then gradually decreasing, and finally dropping rapidly. The crack initiation stress level of the sandstone increased from 0.39 in the control sample (H0) to 0.42 after 12 h of acid treatment (H12), representing an increase of 7.69%. However, with further acid treatment, the crack initiation stress level of the samples decreased rapidly, with a maximum reduction of 40.3%. This trend may be attributed to the initial contact and reaction between the acid and the sandstone, where the acid reacts with certain minerals in the sandstone, slightly enhancing the relative homogeneity of the rock. As the reaction continues, mineral dissolution leads to the dissolution of cementing materials in the sandstone, causing the detachment of fine particles, an increase in the proportion of coarse particles, and an increase in porosity, thereby reducing the crack initiation stress level. This observation is consistent with the findings of Martin^37^ on the crack initiation stress in coarse-grained granite and sandstone.

### Evolution of energy characteristics and acoustic emission response analysis under uniaxial compression

#### Energy characteristics evolution analysis

**Fig. 9:**
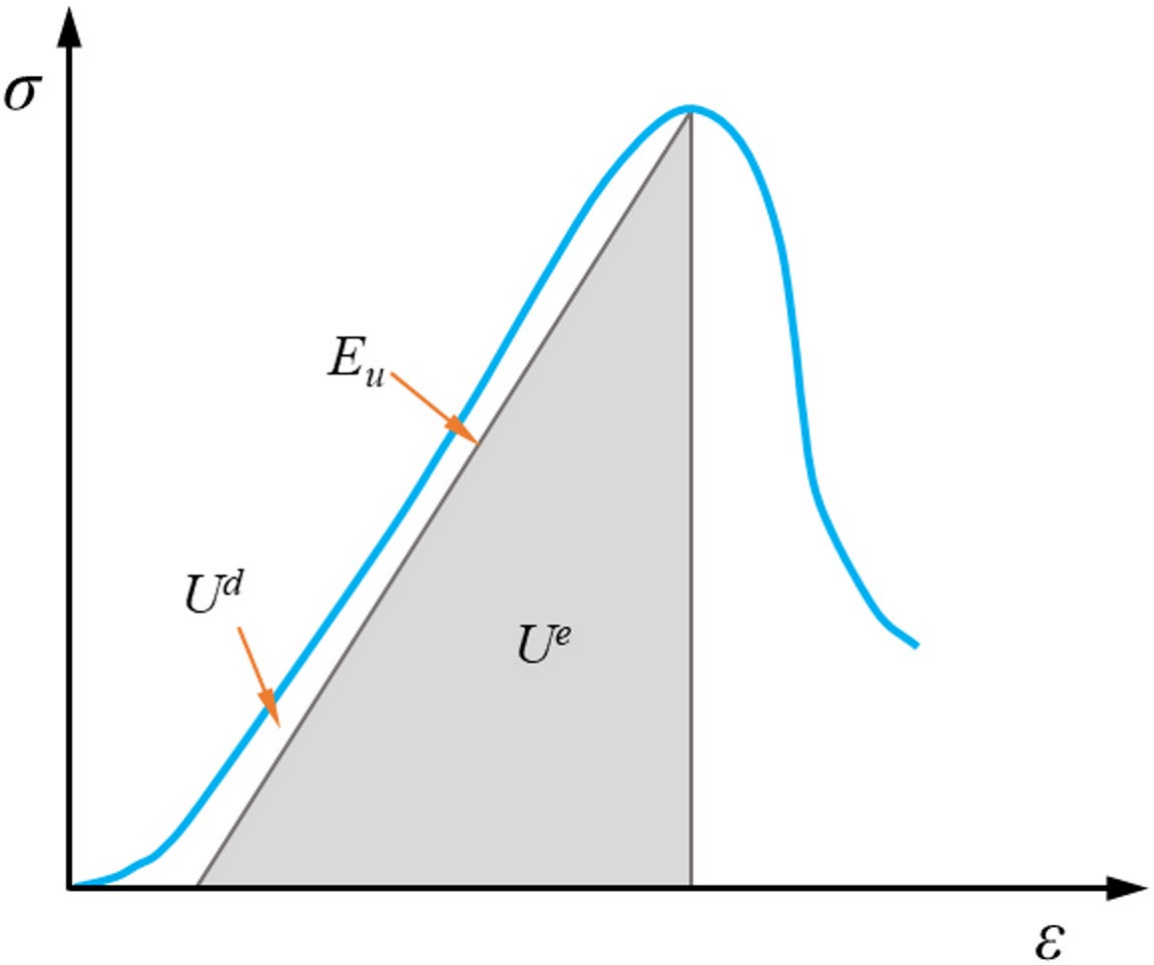
Elastic strain energy and dissipated energy of rock sample.

During the loading process of sandstone samples from the initiation of loading to failure, the work done by the testing machine on the samples (*U*) is mainly composed of two parts: the elastic strain energy stored inside the samples (*U*^d^) and the dissipated energy generated during the compression process (*U*^e^), as shown in Eq. (4). As illustrated in Fig. 9, the shaded area represents the strain energy stored inside the sample during the uniaxial compression process, while the region between the uniaxial compression curve and the shaded area corresponds to the dissipated energy generated in the compression process.

4 $$U={U^e}+{U^d}=\frac{{{\sigma _1}^{2}}}{{2{E_u}}}+{U^d}$$

**Fig. 10:**
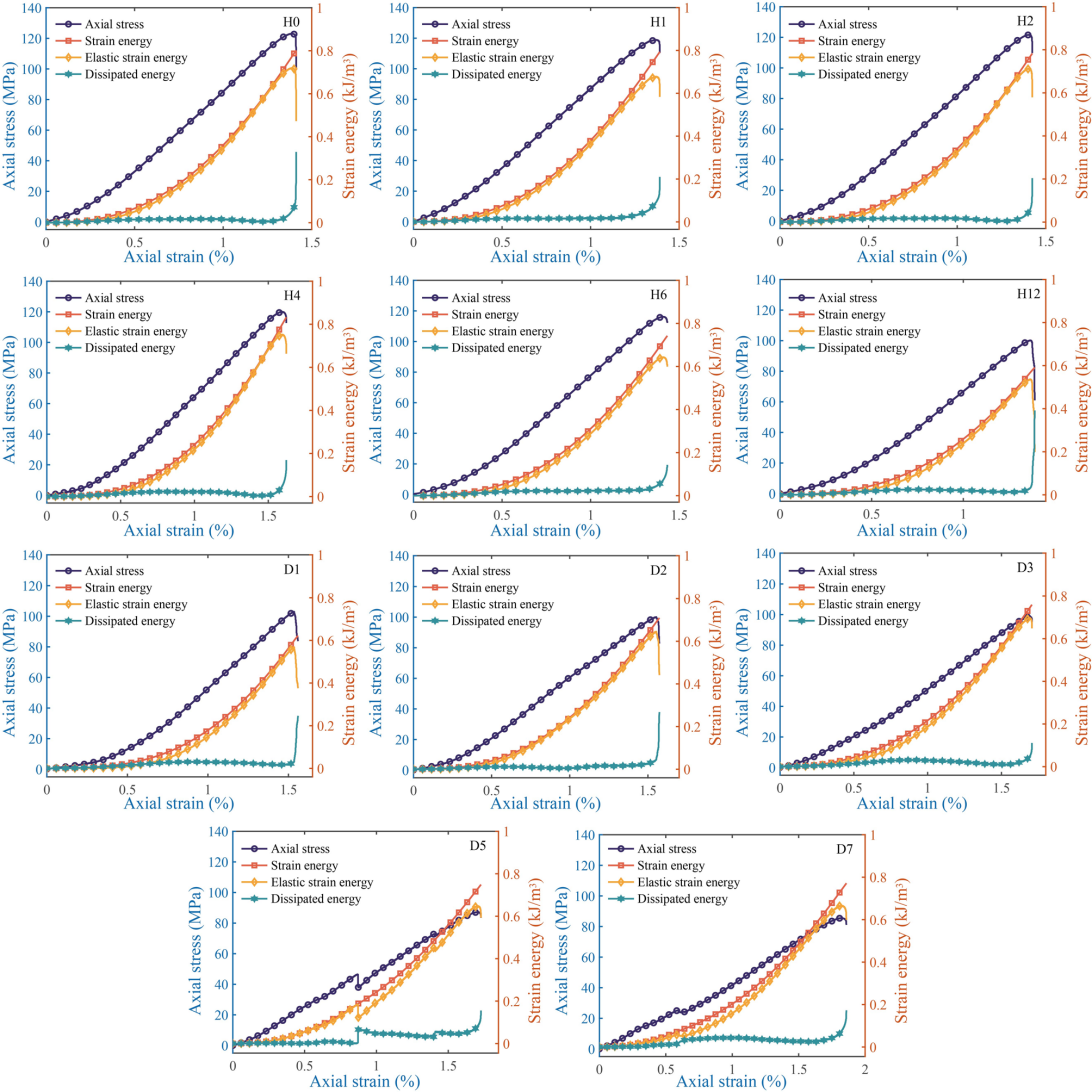
Axial stress-strain and strain energy curves for different acid treatment time.

Sandstone samples treated with acids for different durations exhibit a similar variation pattern in strain energy (Fig. 10). In the initial stage, the dissipated energy of samples H0–D3 is greater than their elastic strain energy. This indicates that during the initial stage of load application, the pores in the sandstone are gradually compacted, and the energy supplied by the testing machine is mainly used for the closure of pores and microcracks as well as frictional losses between rock particles. However, when the acid treatment duration exceeds 120 h (samples D5 and D7), their elastic strain energy is consistently greater than the dissipated energy throughout the entire compression process. This is mainly because the dissolution of some sandstone minerals and cements increases the pores between matrix particles. The load applied by the testing machine is primarily borne by the matrix particles, which reduced the frictional losses between particles, and the work done by the testing machine is mainly stored inside the sample in the form of elastic strain energy.

As the load increases, the axial stress-strain curves of samples D5 and D7 show a sudden drop and a short plateau, respectively. This suggests that after long-term acid treatment, the internal damage of the samples is severe. When the load reaches a certain value, sudden internal failure occurs in the samples, which manifests as a sudden drop in load and a sudden increase in strain. The total input strain energy and elastic strain energy increase approximately exponentially. When the axial strain reaches about 1%, the total input strain energy and elastic strain energy increase approximately linearly. When the axial stress reaches the peak point, the elastic strain energy decreases rapidly.

**Fig. 11:**
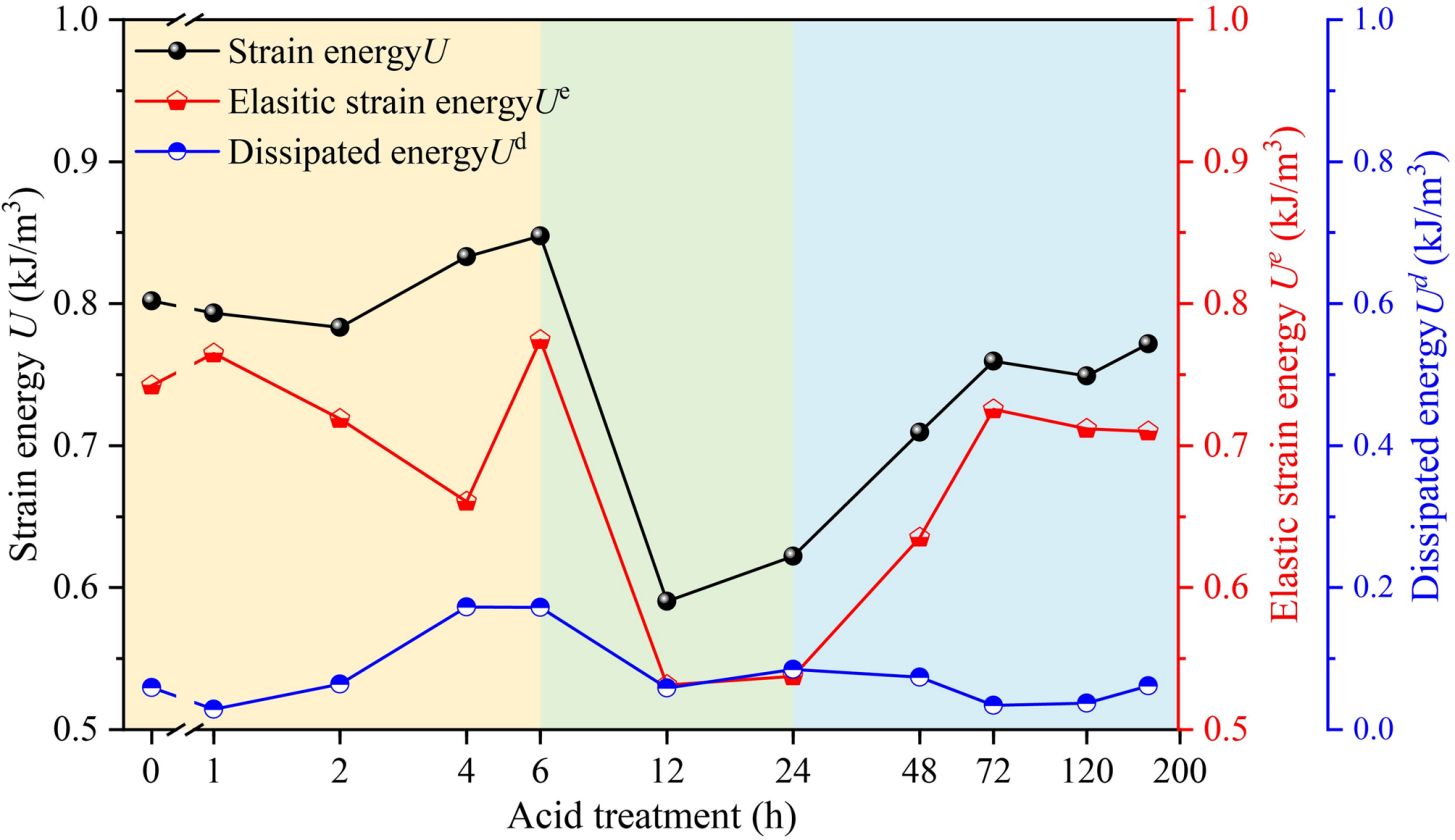
Strain energy of samples with different acid treatment time.

The dissipated energy exhibits a trend of increasing slowly first and then decreasing gradually as the axial strain increases; when the axial stress reaches the peak load, the dissipated energy increases rapidly. This indicates that the work done by the testing machine on the sample is mainly stored inside the sample in the form of elastic strain energy before reaching the peak value. With the increase in acid treatment time, the total input strain energy of the specimens approximately exhibits a variation pattern of increasing slowly first, then decreasing, and finally increasing again (Fig. 11). Among all samples, H12 and D1 have the smallest total input strain energy and elastic strain energy.

#### Acoustic emission response analysis

Figure 3 indicates that acid treatment significantly reduces the uniaxial compressive strength of sandstone, which is related to the dissolution of certain sandstone minerals and cementing materials by the acid solution. This dissolution alters the behavior of particle breakage and inter-particle sliding within the rock under uniaxial compression. Different modes of failure during rock compression exhibit distinct energy release characteristics, which can be investigated by analyzing the parameters of the acoustic emission signals generated during the failure process.

**Fig. 12:**
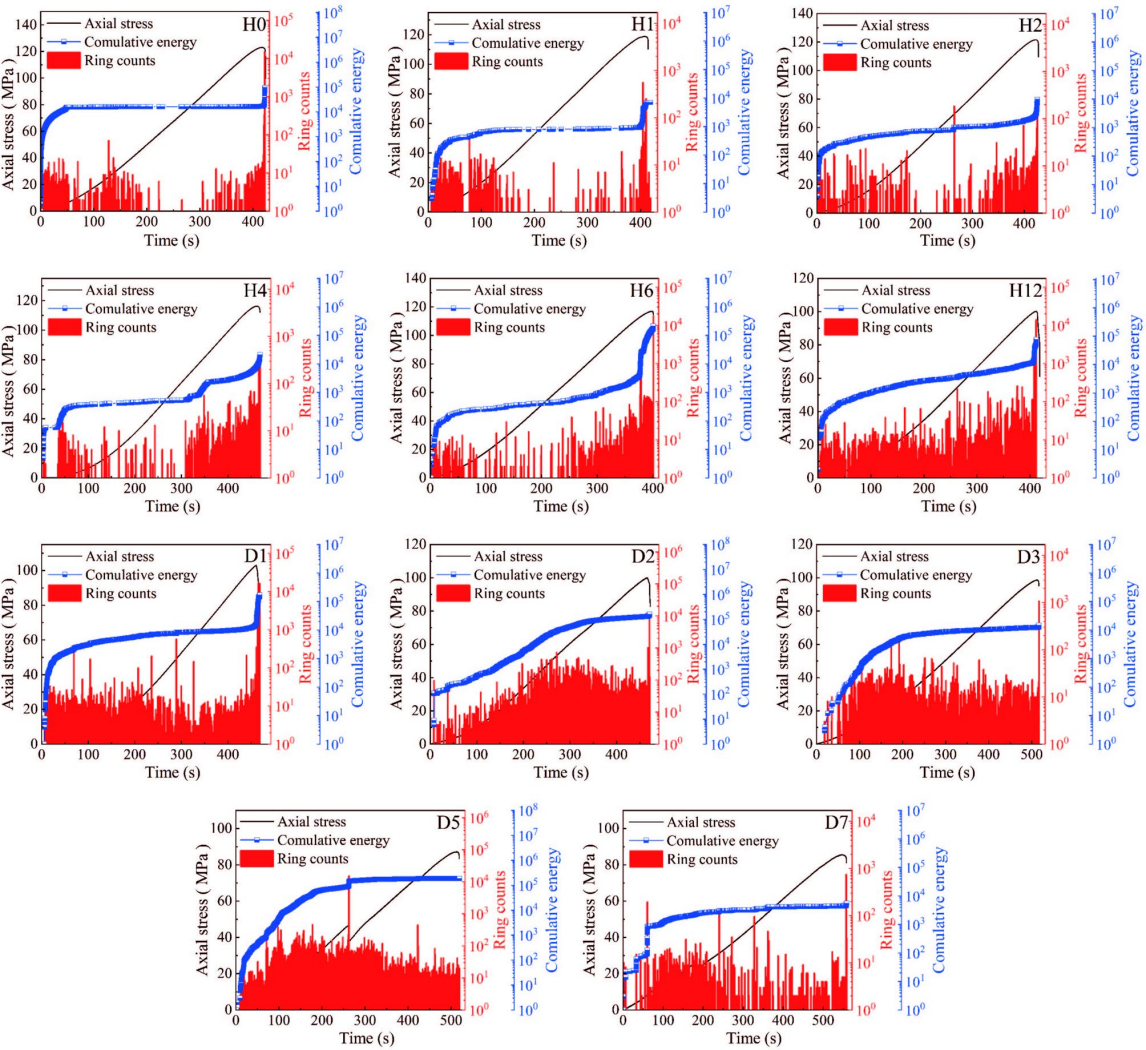
Relationship curves of stress and acoustic emission parameters of sandstone with different acid treatment times.

The acoustic emission characteristics of sandstone samples during uniaxial compression exhibit different changes depending on the acid treatment duration, as shown in Fig. 12. In the untreated sample (H0), a large number of acoustic emission events were recorded early in the loading process. As the acid treatment time increased, samples H1, H2, H4, and H6 also recorded numerous acoustic emission events during the initial compaction stage, but the intensity of ring count occurrences was significantly lower compared to sample H0. This is primarily because the initial contact between the acid solution and the sandstone samples results in a vigorous reaction, where some of the sandstone’s cementing materials dissolve under the corrosive action of the acid. This reduces the contact surface between the rock matrix particles, weakening frictional sliding between sandstone particles in the early stages of uniaxial loading and thereby reducing the intensity of acoustic emission events. During the elastic deformation stage, the rock matrix particles are in close contact, and the deformation of the samples increases steadily with the load, with no new cracks forming inside the samples, leading to fewer acoustic emission events compared to the initial compaction stage. When the axial stress approaches the failure load, acoustic emission events become exceptionally active, with a surge at the peak load.

After 12 h of acid treatment, distinct acoustic emission events were recorded throughout the entire loading process, with increased activity of acoustic emissions. As the acid treatment time increased, the temporal distribution of AE ring counts first increased and then decreased (samples D2, D3, and D5). The active period of acoustic emissions gradually shifted to the early stages of load application. In sample D3, a surge in ring counts was recorded early in the elastic deformation stage, while the maximum ring count in sample D5 occurred in the middle of the loading curve, accompanied by a sharp drop in axial stress. This indicates that under the combined effects of acid pre-treatment and uniaxial compression damage, internal cracks within the sample coalesced, leading to a surge in acoustic emission ring counts.

The cumulative acoustic emission energy of the sandstone samples during the first 24 h of acid treatment can be clearly divided into three stages. In the initial loading phase, the internal pores of the samples were compacted and closed, causing a rapid increase in cumulative energy. After the samples entered the elastic deformation stage, the cumulative energy curve either remained relatively constant (for samples H0, H1, H6, and D1) or exhibited a slow, stepwise increase (for samples H2 and H12). Sample H4 showed a sudden increase in acoustic emission ring count during the elastic deformation stage, indicating that internal cracks within the sample expanded and connected under axial stress, leading to a stepwise increase in the cumulative energy curve. During the failure stage, acoustic emission events became more active as numerous microcracks within the sample expanded and interconnected under axial stress, forming macroscopic fractures. The elastic energy stored within the sample was rapidly released, causing the sample to fail with an accompanying loud sound, and the cumulative energy curve showed a sharp vertical increase.

For samples treated with acid for more than 48 h, the cumulative energy curve can clearly be divided into two stages. In the initial compaction stage, due to pore closure, the cumulative energy curve shows a stepwise increase. Sample D2 exhibits an approximately linear growth trend in the early stages of elastic deformation, with the cumulative energy curve leveling off when the axial stress reaches 71.6 MPa. Upon reaching the failure load, the cumulative energy curve shows a slight vertical increase. For samples D3, D5, and D7, the cumulative energy curve gradually rises during the initial pore compaction stage and remains nearly flat during the elastic deformation stage. However, after reaching the failure load, the cumulative energy values show a sudden increase. This indicates that acid treatment further weakened the cementation between rock matrix particles, increasing the frictional sliding between these particles during the pore compaction stage, as reflected by the continuous increase in cumulative energy during this phase.

## Discussion

Rock brittleness is an important indicator for evaluating reservoir fracability. It is generally believed that reservoir brittleness is highly correlated with the mineral composition of the rock, and a mineral composition method has been established to evaluate rock brittleness based on the content of the primary brittle minerals in the rock. However, this method overlooks the impact of sedimentation on reservoir rocks and relies heavily on obtaining the content of brittle minerals in the rock. Conducting various mechanical tests on reservoir rock samples to obtain the full stress-strain curve and then deriving characteristic parameters to evaluate rock brittleness is a more direct and objective approach. However, for high-strength and high-brittleness rock samples, it is challenging to obtain the post-peak portion of the full stress-strain curve. For tight sandstone, the pre-peak portion of the stress-strain curve primarily reflects the elastic and inelastic deformation properties of the rock, specifically the initiation and propagation of cracks, while the post-peak portion mainly reflects the rock’s load-bearing capacity after failure. Reservoir modification in oil and gas fields focuses more on the initiation and propagation of rock cracks, with less emphasis on the post-fracture morphology of the rock. Therefore, considering the stress-strain changes of the rock from crack propagation initiation to peak failure in the pre-peak section of the stress-strain curve is of great significance for the fracturing modification of tight oil and gas reservoirs.

Due to the influence of tectonic stress, tight sandstone reservoirs generally exhibit high fracture pressures, making it difficult to successfully fracture the reservoir using existing techniques and equipment. Reservoir acid treatment is an effective method to reduce fracture pressure. Existing studies have focused more on analyzing the impact of acid treatment on the pore structure and strength characteristics of rocks^37^, with less attention given to the effect of acid treatment on the brittleness of reservoir rocks. As shown in Fig. 8, with the increase in acid treatment time, the brittleness index of tight sandstone initially increases and then decreases, reaching its maximum between 12 and 24 h of acid treatment.

The evolution of brittleness is intrinsically linked to the microstructural alterations caused by acid-rock reactions. XRD results (Fig. 2) confirm the dissolution of quartz, feldspar, and chlorite, which constitute the primary load-bearing framework and cementing materials. The mass dissolution rate (Fig. 4) indicates rapid initial reaction followed by stabilization, aligning with the stepwise decrease in UCS. During the initial stage of acid treatment, acid preferentially dissolves soluble minerals and weak cementing materials at grain boundaries and within pre-existing microdefects. This contributes to an increase in the overall homogeneity of the rock and the formation of a uniform stress distribution. Meanwhile, the generation of new micropores and the enlargement of existing ones can act as stress concentrators, promoting crack initiation under lower global stress, but may also favor the formation of scattered microcracks rather than localized macrocracks. The peak brittleness observed in specimens subjected to 12–24 h of acid treatment indicates that uniform stress distribution plays a dominant role, which requires higher stress to trigger crack propagation. Acoustic emission results show that more high-intensity signals appear in the elastic stage after 12 h of acid treatment, suggesting the formation of more active yet scattered microcracks under acidization.

However, with prolonged acid treatment (> 24 h), dissolution becomes more extensive, resulting in significant detachment of fine-grained matrix particles and a substantial increase in porosity, which severely weakens the granular framework of the rock matrix. In addition, the increased porosity and reduced intergranular cohesion significantly lower the crack initiation stress. Under uniaxial compression, the failure mode shifts toward greater frictional sliding between loosened grains and pore collapse, dissipating more energy in a non-brittle manner. This is corroborated by the AE results for samples D2, D3, D5, and D7, where the active period of acoustic emissions shifts to earlier loading stages, and the failure is not accompanied by a sharp AE surge, indicating a more ductile, compactive failure mechanism rather than a brittle, tensile-dominated fracture. This also suggests that prolonged acid treatment increases frictional sliding between the sandstone matrix particles, which is unfavorable for the propagation of long fractures. Additionally, under 24 h of acid treatment (sample D1), with the increase of axial load, there is a dense occurrence of high-energy acoustic emission signals during the pore compaction and elastic deformation stages of the sandstone, indicating pronounced brittle failure characteristics, which facilitates the formation and propagation of fractures.

Currently, fracturing remains the primary method for enhancing production in oil and gas reservoirs. For tight sandstone reservoirs with high fracture pressure, effectively reducing the reservoir fracture pressure and forming a complex fracture network are crucial for successful reservoir modification and development. Existing acid treatment techniques for reducing fracture pressure often overlook the impact of acid treatment duration on reservoir modification and development. The rock brittleness index evaluation method proposed in this study allows for accurate measurement of the changes in rock brittleness under different acid treatment durations. This provides a basis for selecting a reasonable acid treatment duration for reducing fracture pressure in high-pressure reservoirs, ensuring that the reservoir fracture pressure is lowered while maintaining sufficient brittleness to achieve the desired fracture network modification.

## Conclusion

This study proposes a novel brittleness index evaluation method based on the radial strain response to determine crack initiation stress and considering the stress-strain segment from crack initiation to peak stress. This method is particularly advantageous for assessing the time-dependent effects of acidization on tight sandstone, as it focuses on the crack propagation phase most relevant to hydraulic fracturing, unlike methods relying on post-peak behavior which is often unobtainable for high-strength brittle rocks.

The results show that the brittleness index reaches its maximum within 12–24 h of acid treatment. This suggests that an optimal acid treatment duration exists: too short a treatment may not sufficiently reduce fracture pressure, while too long a treatment may excessively reduce brittleness, hindering the formation of a complex fracture network. Therefore, this research offers a quantitative basis for optimizing acid pre-treatment schedules in high fracture pressure tight sandstone reservoirs to achieve both reduced breakdown pressure and enhanced fractability.

## Study limitations and future prospects

The experiments were conducted under uniaxial compression, which does not account for the confining pressure (in-situ stress) prevalent in subsurface reservoirs. Confining pressure is known to suppress crack propagation, promote pore collapse, and can significantly alter the brittle-ductile transition boundary of rocks. The observed brittleness trends under uniaxial conditions might differ under triaxial stress states. Future work should involve triaxial compression tests​ to investigate how different confining pressures modulate the acid-induced brittleness changes reported here. In addition, the use of static immersion in this study differs from the dynamic flow conditions in field acidizing operations. Dynamic flow can lead to more complex dissolution patterns, such as the formation of wormholes or face dissolution, which may create heterogeneous weakening paths rather than the relatively uniform surface dissolution likely occurring in static tests. This difference could affect the spatial distribution of brittleness alteration and the overall effectiveness of the treatment. Future research should consider core-flood experiments​ with flowing acid to better simulate field conditions.

## Data Availability

The datasets generated during the current study are available from the corresponding author on reasonable request.
